# A study of the influence of the T2DL.2DS-2SS translocation and the 5S(5D) substitution from Aegilops speltoides on breeding-valuable traits of common wheat

**DOI:** 10.18699/vjgb-24-57

**Published:** 2024-09

**Authors:** R.O. Davoyan, I.V. Bebykina, E.R. Davoyan, A.N. Zinchenko, Y.S. Zubanova, D.M. Boldakov, V.I. Basov, E.D. Badaeva, I.G. Adonina, E.A. Salina

**Affiliations:** National Center of Grain named after P.P. Lukyanenko, Krasnodar, Russia; National Center of Grain named after P.P. Lukyanenko, Krasnodar, Russia; National Center of Grain named after P.P. Lukyanenko, Krasnodar, Russia; National Center of Grain named after P.P. Lukyanenko, Krasnodar, Russia; National Center of Grain named after P.P. Lukyanenko, Krasnodar, Russia; National Center of Grain named after P.P. Lukyanenko, Krasnodar, Russia; National Center of Grain named after P.P. Lukyanenko, Krasnodar, Russia; N.I. Vavilov Institute of General Genetics Russian Academy of Sciences, Moscow, Russia; Institute of Cytology and Genetics of the Siberian Branch of the Russian Academy of Sciences, Novosibirsk, Russia; Institute of Cytology and Genetics of the Siberian Branch of the Russian Academy of Sciences, Novosibirsk, Russia

**Keywords:** Triticum aestivum, Aegilops speltoides, introgressive lines, chromosomes, translocations, molecular markers, disease resistance, productivity and technological qualities of grain, Triticum aestivum, Aegilops speltoides, интрогрессивные линии, хромосомы, транслокации, молекулярные маркеры, устойчивость к болезням, продуктивность и технологические качества зерна

## Abstract

The use of the gene pool of wild relatives for expanding the genetic diversity of common wheat is an important task of breeding programs. However, the practical application of common wheat lines with alien genetic material is constrained by the lack of information on chromosomal rearrangements and the negative impact of the transferred material on agronomically important traits. This research is aimed at studying 14 introgression lines with the T2DL.2DS-2SS translocation and the 5S(5D) substitution from Aegilops speltoides obtained from crossing common wheat varieties (Aurora, Krasnodarskaya 99, Nika Kubani) with the genome-substituted form Avrodes (BBAASS). Hybrid lines with different combinations of T2DL.2DS-2SS and T1BL.1RS translocations and 5S(5D) substitution were characterized by resistance to leaf and yellow rusts, productivity components and technological qualities of grain. The assessment of the varieties’ resistance to rust diseases showed that Krasnodarskaya 99, Nika Kubani and the Aurora variety, which is a carrier of the T1BL.1RS translocation, are highly susceptible to diseases, while the presence of the T2DL.2DS-2SS translocation and the 5S(5D) substitution, both together and separately, provides resistance to fungal pathogens. The analysis of the lines using markers designed for known resistance genes of Ae. speltoides did not reveal the presence of the Lr28, Lr35 and Lr51 genes in the lines. The results suggest that the genetic material of Ae. speltoides transferred to chromosomes 2D and 5D contains new resistance genes. To determine the effect of the T2DL.2DS-2SS translocation and the 5S(5D) substitution on the productivity and technological qualities of grain, the lines were assessed by weight of 1000 grains, grain weight and number of ears per 1 m2, by protein and gluten content, gluten quality and general baking evaluation. A positive effect was determined upon the weight of 1000 grains, protein and gluten content. There were no significant differences in other characteristics. The T2DL.2DS-2SS translocation and the 5S(5D) substitution did not have a negative effect on the productivity and technological quality of grain, and are of interest for breeding practice.

## Abstract (ru)

Использование генофонда диких сородичей для расширения генетического разнообразия мягкой пшеницы является актуальным направлением селекции. Однако практическое применение линий мягкой пшеницы с чужеродным генетическим материалом сдерживается ввиду отсутствия информации о хромосомных перестройках и их влиянии на важные хозяйственные признаки. Целью настоящей работы было изучение 14 интрогрессивных линий с транслокацией T2DL.2DS-2SS и замещением 5S(5D) от Aegilops speltoides, полученных от скрещивания сортов мягкой пшеницы Аврора, Краснодарская 99, Ника Кубани с геномно-замещенной синтетической формой Авродес (BBAASS). Гибридные линии с различным сочетанием транслокаций T2DL.2DS-2SS и T1BL.1RS и замещения 5S(5D) были охарактеризованы по устойчивости к листовой и желтой ржавчинам, компонентам продуктивности и технологическим качествам зерна. Оценка устойчивости сортов к ржавчинным болезням показала, что Краснодарская 99, Ника Кубани и сорт Аврора (носитель транслокации T1BL.1RS) высоковосприимчивы к болезням, тогда как наличие транслокации T2DL.2DS-2SS и замещения 5S(5D) как совместно, так и по отдельности обеспечивает устойчивость линий к грибным патогенам. Анализ линий с помощью маркеров, разработанных для известных генов устойчивости от Ae. speltoides, не выявил в линиях присутствия генов Lr28, Lr35 и Lr51. Полученные результаты позволяют предположить, что генетический материал Ae. speltoides в хромосомах 2D и 5D содержит новые гены устойчивости. Для определения влияния транслокации T2DL.2DS-2SS и замещения 5S(5D) на продуктивность и технологические качества зерна проведено изучение линий по массе 1000 зерен, массе зерна и количеству колосьев с 1 м2, содержанию белка и клейковины, качеству клейковины и общей хлебопекарной оценке. Установлен положительный эффект по массе 1000 зерен, содержанию белка и клейковины. По остальным признакам существенных различий не найдено. Транслокация T2DL.2DS-2SS и замещение 5S(5D) не оказывают негативного влияния на продуктивность и технологические качества зерна и представляют интерес для селекционной практики.

## Introduction

The basis of breeding, including that of such an important agricultural crop as common wheat (Triticum aestivum L.), is sufficient genetic diversity. The intensification of the breeding process and the widespread distribution of varieties of the same type have led to significant genetic erosion, especially of disease resistance genes. An effective way to solve this problem is to use the gene pool of numerous species and genera related to common wheat (Knott, 1987; Friebe et al., 1996).

One of the wild relatives most widely used as a source of disease resistance is the species Aegilops speltoides Tausch (Manisterski et al., 1988; Kerber, Dyck, 1990). This species has given wheat genes for resistance to leaf rust – Lr28, Lr35, Lr36, Lr47, Lr51 and Lr66, to stem rust – Sr32, Sr39, Sr47, to powdery mildew – Pm12, Pm32 (McIntosh et al., 2013). Ae. speltoides is also characterized by its high protein content and the ability to stimulate homeologous chromosome conjugation (Dvorak, 1972). However, due to a negative impact on other economically valuable traits, introgression from this species has not been widely used in breeding practice (McIntosch et al., 1995; Helguera et al., 2005; Song et al., 2007; Brevis et al., 2008). It should be noted that the negative effect of alien introgression may depend both on the negative influence of the genetic material of the wild relative transferred along with the target gene, and on the genotypic environment of the recipient variety (Hoffann, 2008; Leonova, Budashkina, 2016).

At the “P.P. Lukyanenko National Grain Center”, the genome substitution form Avrodes (BBAASS) has been developed, which is used as a “bridge” for the transfer of genetic material from Ae. speltoides to common wheat (Zhirov, Ternovskaya, 1984; Davoyan R.O. et al., 2012). This form exhibits high resistance to leaf rust (Puccinia triticina Eriks.), yellow rust (Puccinia stiifomis West.), powdery mildew (Blumeria graminis f. sp. tritici) and is characterized by a high protein content (Davoyan R.O. et al., 2018). This form has been involved in obtaining a large set of introgressive lines of common wheat, differing in the complex of morphological, biological and economically valuable traits, in the form of transmission of genetic material from Ae. speltoides (Davoyan R.O. et al., 2017).

Using the methods of differential chromosome staining (C- banding) and fluorescent in situ hybridization (FISH), it was found that introgressions affected mainly the chromosomes of the D genome. This is explained by the fact that in the synthetic form of Avrodes it is the D genome of common wheat that is replaced by the S genome of Ae. speltoides. Moreover, most of the studied lines are characterized by the T2DL.2DS-2SS translocation and the 5S(5D) substitution. To determine the breeding value of the resulting translocations and substitutions from Ae. speltoides, a comprehensive study of introgression lines based on economically important traits is required

This research is aimed at the study of the impact of the T2DL.2DS-2SS translocation and the 5S(5D) substitution from Ae. speltoides on productivity, grain quality and resistance to fungal diseases of three varieties of common wheat of different origin.

## Materials and methods

The material for the study was 14 introgressive lines of common wheat obtained from crossing the synthetic form Avrodes with the varieties susceptible to leaf and yellow rust, bred by the “P.P. Lukyanenko National Grain Center”: Aurora, Krasnodarskaya 99 and Nika Kubani. Lines based on the Krasnodarskaya 99 and Aurora varieties were obtained previously (Davoyan R.O. et al., 2017) and were selected within the framework of this work for researching the presence of the T2DL.2DS-2SS translocations and the 5S(5D) substitution. The lines obtained on the basis of the Nika Kubani variety were characterized by cytological methods as part of this research.

Differential staining of chromosomes (C-banding) was carried out at the “N.I. Vavilov Institute of General Genetics, RAS” using a method developed in the Laboratory of Functional Chromosome Morphology of the “V.A. Engelhardt Institute of Molecular Biology, RAS” (Badaeva et al., 1994). Fluorescence in situ hybridization (FISH) was carried out at the “Institute of Cytology and Genetics, SB RAS” according to a previously published method (Salina et al., 2006) using probes: Spelt1 (Salina et al., 2004) to identify the genetic material of Ae. speltoides in the studied lines; pSc119.2 (Bedbrook et al., 1980) and pAs1 (Rayburn, Gill, 1986) to identify wheat and aegilops chromosomes (Badaeva et al., 1996; Schneider et al., 2003). The work was carried out at the Center for Microscopic Analysis of Biological Objects of the SB RAS (Novosibirsk).

Infestation of the lines was carried out under field conditions, with yellow rust in the booting phase, and with leaf rust in the boot-heading phase. In both cases, a mixture of uredospores collected from different varieties of wheat was used. The assessment was carried out when the most susceptible and late-ripening recipient variety, Aurora, reached maximum susceptibility rates (reaction type 4, degree of damage 60 %). The type of plant reaction to infection with leaf rust was determined according to the scale of E.B. Mains and H.S. Jackson (1926); to yellow rust, according to the scale of G. Gassner and U.W. Straib (1934). Plants with an intermediate type of reaction from 0 to 1 were designated as 01. The degree of plant damage was assessed using the modified Cobb scale (Peterson et al., 1948). Plants with a reaction type from 0 to 2 and a degree of damage from 0 to 20 % were classified as resistant.

DNA was isolated from 5–7-day-old etiolated wheat seedlings according to the method of J. Plaschke et al. (1995). Identification of the Lr28, Lr35 and Lr51 genes was carried out using PCR. Markers were selected according to the publication data; their names and amplification conditions are presented in Table 1.

**Table 1.**
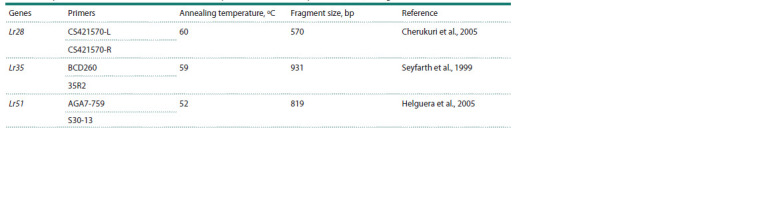
Amplification conditions, names and sources of primers used to identify the Lr28, Lr35, Lr51 genes

A 25 μL reaction mixture contained 1× buffer for Taq-DNA polymerase (50 mM KCl, 20 mM Tris-HCl, pH 8.4, 2–5 mM MgCl2, 0.01 % Tween-20), 2 mM MgCl2, 0.2 mM of each dNTP, 12.5 mM of each primer, 50 ng DNA and 1 unit of Taq polymerase. Amplification was carried out according to the conditions given in Table 1. PCR products for the Lr28 and Lr35 genes were separated using electrophoresis in a 1.8 % agarose gel with 0.5× TBE buffer; in the case of the Lr51 gene, a 3 % agarose gel was used with MS-12 agarose, Molecular Screening “diaGene” with increased clarity of fragment separation. DNA marker M24 100 bp “SibEnzyme” was used as a molecular weight marker. Gels were stained with ethidium bromide and photographed under ultraviolet light using an Infiniti 1000 photobox.

To characterize the lines by productivity, the weight of 1000 grains, grain weight and the number of ears per plot were determined. The plot area was 1 m2, there were four replications. The technological qualities of grain and flour were studied in the Department of Technology and Biochemistry of Grain of the “P.P. Lukyanenko National Grain Center” according to the methods of the State Variety Testing of Agricultural Crops (1988). Statistical processing of the obtained results was carried out using the AGROS-2.10 program.

## Results

To determine the breeding value of the T2DL.2DS-2SS translocation and the 5S(5D) substitution from Ae. speltoides, a study was carried out on 14 introgressive lines obtained with the participation of three varieties susceptible to leaf and yellow rust: Aurora, Krasnodarskaya 99 and Nika Kubani. The characteristics of the lines concerning introgressions and resistance to leaf and yellow rusts are given in Table 2.

**Table 2.**
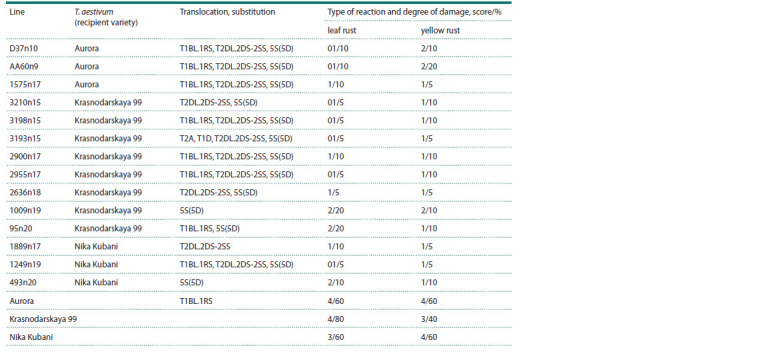
The characteristics of the T. aestivum/Avrodes lines concerning introgressions and resistance to leaf and yellow rusts

**Fig. 1.**
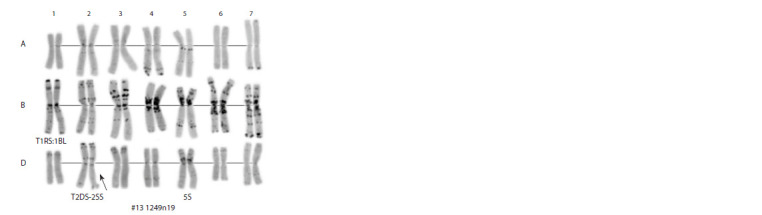
Differentially stained karyotype of line 1249n19.

**Fig. 2.**
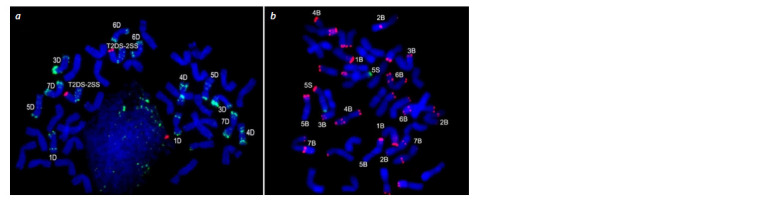
FISH results on metaphase chromosomes of lines: (a) 1889n17 with probes pAs1 (green) and Spelt1 (red); (b) 493n20 with probes pSc119.2 (red) and Spelt1 (green).

The majority of the presented lines are characterized by a combination of the T2DL.2DS-2SS translocation and the 5S(5D) chromosomal substitution (Table 2, Fig. 1). Also, a significant number of the lines have the T1BL.1RS translocation (Table 2, Fig. 1), obtained from the synthetic form of Avrodes. In line 1889n17, a single T2DL.2DS-2SS translocation was detected (Fig. 2a). Only a 5S(5D) chromosomal substitution was detected in lines 1009n19 and 493n20 (Fig. 2b).

Recipient varieties Aurora, Nika Kubani and Krasnodarskaya 99 are susceptible to leaf and yellow rust. The T2DL.2DS-2SS translocation and the 5S(5D) substitution, both together and separately, provide line resistance to these pathogens (Table 2). Line 1889n17 with the T2DL.2DS-2SS translocation exhibits higher resistance to leaf rust (reaction type 1, severity of damage 10 %) compared to lines 1009n19, 95n20 and 493n20 with the 5S(5D) substitution

**Fig. 3.**
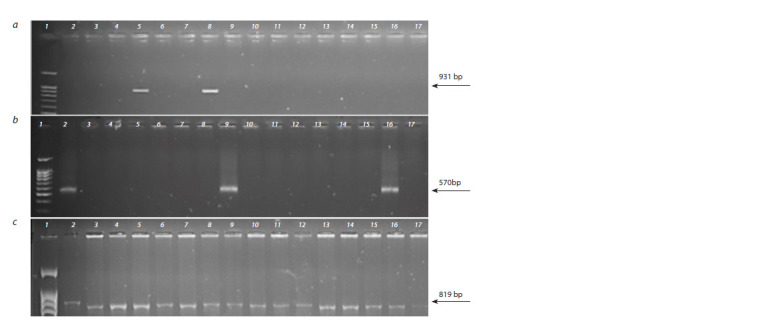
Electropherograms of amplification products using primers to diagnostic markers linked to genes: a) Lr35 (1 – length marker, 2 – Aurora, 5 – TcLr35, 8 – Avrodes; 2–4, 6, 7, 9–17 – introgression lines); b) Lr28 (1 – length marker, 2 – TcLr28, 9, 16 – Avrodes, 3 – Aurora, 4–8, 10–15, 17 – introgression lines); c) Lr51 (1 – length marker, 2 – Avrodes, 3 – Aurora, 4–17 – introgression lines).

Since one of the main objectives was the transfer of resistance to leaf rust from the synthetic form Avrodes, genes for resistance to this disease were identified using DNA markers. Among the known, identified leaf rust resistance genes derived from Ae. speltoides, the effective gene Lr35 was found in Avrodes (Davoyan E.R et al., 2012) (Fig. 3a), as well as the genes Lr28 and Lr51 (Fig. 3b and 3c, respectively). Since the absence of the Lr28 and Lr35 genes in the AA60n9 line was previously determined (Davoyan R.O. et al., 2017), in this research this line was studied for the presence of only the Lr51 gene. There were no Lr28, Lr35 and Lr51 genes found in the studied lines (Fig. 3a, 2–4, 6, 7, 9–17; Fig. 3b, 4–8, 10–15, 17; 3c, 4–17).

To determine the breeding value of the T2DL.2DS-2SS translocation and the 5S(5D) substitution, the lines were assessed for productivity components and technological qualities of grain and flour.

Productivity was determined by the weight of 1000 grains, the weight of grains and the number of ears per 1 m2 (Table 3). In the lines obtained with the participation of the Aurora variety as a recipient, a significant excess in the weight of 1000 grains was revealed. The highest value for this indicator had line 1575n17 (41.7 g). There were no significant differences in the number of formed ears per 1 m2. In terms of grain weight per 1 m2, lines D37n10 and AA60n9 were at the same level, and line 1575n17 was significantly higher than the Aurora variety.

**Table 3.**
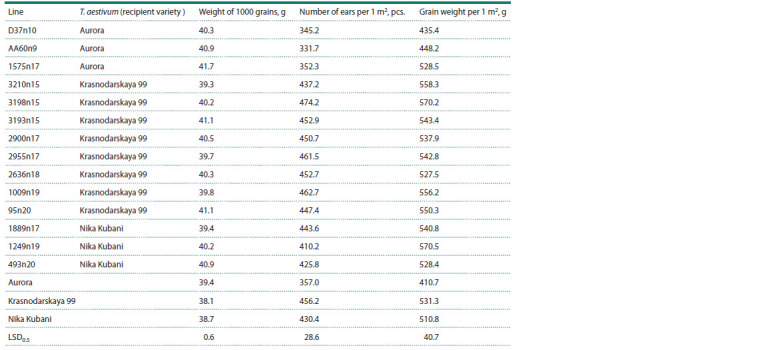
Productivity components of introgressive lines of T. aestivum/Avrodes Notе. LSD – least significant difference.

All lines obtained with the participation of the Krasnodarskaya 99 variety reliably exceeded it in the weight of 1000 grains. There were no significant differences in the number of ears per 1 m2 and grain weight.

Line 1249n19 significantly exceeded the recipient variety Nika Kubani in the weight of 1000 grains (40.2 g) and the weight of grain per 1 m2 (570.5 g). The weight of 1000 grains for lines 1889n17 and 493n20 was also higher than that of Nika Kubani. The differences in the number of ears and grain weight per 1 m2 were insignificant.

Important features that limit the use of lines carrying alien genetic material in breeding practice are the technological characteristics of grain. The lines obtained with the participation of the Aurora variety as a recipient have similar characteristics of protein and gluten content, gluten quality and general baking assessment (Table 4).

**Table 4.**
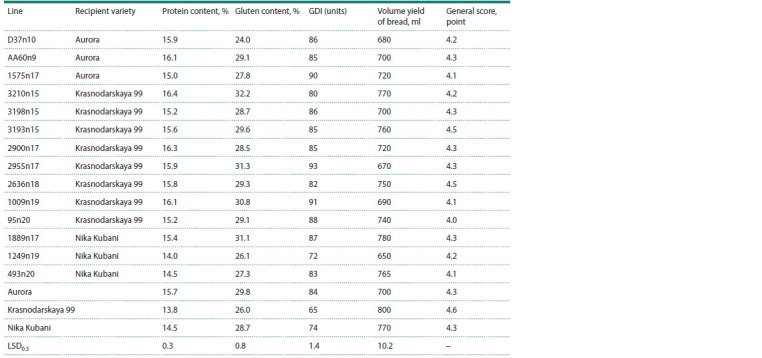
Technological characteristics of introgressive lines of T. aestivum/Avrodes Notе. GDI – gluten deformation index.

The transfer of the T2DL.2DS-2SS translocation and the chromosomal 5S(5D) substitution to the Krasnodarskaya 99 variety contributed to an increase in protein and gluten content in the lines (Table 4). The excess protein content in lines 3210n15, 2955n17, 2636n18 and 1009n19 ranged from 2 to 3 %. At the same time, it should be noted that all lines have higher GDI values and correspond to the second group of GOST in terms of gluten quality. The lines are also inferior to the Krasnodarskaya 99 variety in terms of volumetric bread yield and overall baking rating. Lines 1249n19 and 493n20 have approximately the same performance as the recipient variety Nika Kubani. Line 1889n17 exceeds the recipient variety in protein and gluten content, but is inferior to it in gluten quality (Table 4).

## Discussion

The transfer of economically valuable genes from the gene pool of numerous related species and genera to common wheat remains an effective way of solving current breeding problems

The purpose of using the synthetic form Avrodes, first of all, was the transfer of new genes for resistance to diseases, in particular to leaf rust, from Ae. speltoides to common wheat. Currently, the catalog of wheat gene symbols includes six resistance genes transmitted from this species: Lr28, Lr35, Lr36, Lr47, Lr51 and Lr66 (McIntosh et al., 2013), respectively localized in common wheat chromosomes 4A, 2B, 6B, 7A, 1B and 3A (Friebe et al., 1996). Additionally, I.G. Adonina et al. (2012) characterized the T5BS.5BL-5SL translocation from Ae. speltoides with an effective gene designated LrASP5.

Our molecular genetic analysis did not reveal in the studied wheat lines the presence of effective resistance genes Lr28, Lr35 and Lr51 present in the synthetic Avrodes. We found that the T2DL.2DS-2SS translocation and the 5S(5D) substitution from Ae. speltoides, both together and separately, provide wheat lines with resistance to leaf rust. At the same time, line 1889n17 with the T2DL.2DS-2SS translocation is characterized by higher resistance to leaf rust (reaction type 1) than lines with only the 5S(5D) substitution (reaction type 2) (Table 2). None of the previously transferred known leaf rust resistance genes are located on chromosomes 2D and 5D. According to S.N. Sibikeev et al. (2015), the 2D/2S translocation is carried by lines L195 and L200, which are resistant to leaf and stem rust. Due to the lack of these lines at our disposal, we were unable to clarify the identity of these leaf rust resistance genes with the genes present in the lines we obtained.

It should also be noted that our lines with the T2DL.2DS- 2SS translocation and the 5S(5D) substitution are resistant to yellow rust, which is one of the most common wheat diseases. Although until the end of the 1960s it had no economic significance on the territory of Russia, since 1990, in the south, primarily in the Krasnodar region, the yellow rust pathogen has had a tendency of expanding its range, and the damage to some varieties of winter wheat against a natural infectious background has reached 90 % (Volkova et al., 2020). At the same time, not a single yellow rust resistance gene transferred to the wheat genome from Ae. speltoides (McIntosh et al., 2013) is registered in the catalog of gene symbols. Thus, our results indicate the possible transfer of new resistance genes to common wheat from this species. Additional research is necessary to test this assumption.

When transferring alien genetic material, along with useful traits (disease resistance, high protein content, etc.), introgressions often have a negative impact on the productivity and technological characteristics of grain. For this reason, a number of alien translocations have not found wide application in breeding practice. Thus, of the abovementioned six resistance genes to leaf rust, only the Lr28 and Lr47 genes are used in practical breeding (Leonova, 2018). At the same time, introgression lines with genetic material of Ae. speltoides can combine disease resistance with productivity and good technological qualities of grain and flour (Lapochkina et al., 1996; Sibikeev et al., 2015; Davoyan R.O. et al., 2017).

Based on our results (Table 3), we can conclude that the presence of the T2DL.2DS-2SS translocation and even the 5S(5D) chromosomal substitution in the lines does not have a negative effect on the elements of productivity. Two lines, 1575n17 and 1249n19, significantly exceed the recipient varieties Aurora and Nika Kubani, respectively, in terms of grain weight per 1 m2. In the remaining lines, no significant differences were found either in the number of ears per 1 m2 or in grain weight. A positive effect of the T2DL.2DS-2SS translocation and the 5S(5D) substitution on the weight of 1000 grains was determined. Almost all the lines we studied exceeded the recipient varieties for this trait, while, for example, in the work of N.V. Petrash et al. (2016), a decrease in the weight of 1000 grains was noted in all introgressive lines, regardless of chromosomal localization (chromosomes 5ВL, 6ВL and 7D) of alien chromatin.

The study of the technological characteristics of grain and flour also did not reveal a negative effect of the T2DL.2DS- 2SS translocation and the 5S(5D) substitution (Table 4). There were no significant differences in protein and gluten content, gluten quality and overall baking rating between the Aurora variety and the lines obtained on its basis. The lines obtained with the Krasnodarskaya 99 variety, in comparison with it, have higher levels of protein and gluten content and, despite a slight deterioration in the quality of gluten (second group of GOST), in general, they received a fairly high baking rating. The technological characteristics of the Nika Kubani/Avrodes lines are similar to those for the recipient variety Nika Kubani.

The manifestation of traits in introgressive lines depends not only on the alien genetic material present in them, but also on the genotypic environment of the recipient variety. In our studies, the nature of the manifestation of the T2DL.2DS-2SS translocation and the 5S(5D) substitution was studied on the genetic background of three common wheat varieties of different origins. The lines combine disease resistance with good indicators of productivity, grain and flour quality, regardless of the recipient variety

## Conclusion

Thus, we can conclude that the resulting new translocation T2DL.2DS-2SS and the substitution 5S(5D) from Ae. speltoides can contribute to the improvement of common wheat, in particular in terms of disease resistance, protein and gluten content, as well as weight of 1 000 grains, and are of interest for breeding practice.

## Conflict of interest

The authors declare no conflict of interest.
